# Therapeutic Biomaterials for Chronic Osteomyelitis: Time–Space–Control Strategies for Infection Control and Bone Repair—A Narrative Review

**DOI:** 10.3390/jfb17030142

**Published:** 2026-03-12

**Authors:** Jinqiu Tian, Qi Meng, Peixun Zhang

**Affiliations:** 1 Department of Trauma and Orthopaedics, Peking University People’s Hospital, Beijing 100044, China; 20259023022@stumail.hbu.edu.cn (J.T.); 2511110399@bjmu.edu.cn (Q.M.); 2 National Centre for Trauma Medicine, Beijing 100044, China; 3 Key Laboratory of Trauma and Neural Regeneration, Peking University, Beijing 100044, China; 4 Beijing Laboratory of Trauma and Nerve Regeneration, Peking University, Beijing 100044, China; 5 Clinical Medical College, Hebei University, Baoding 071000, China; 6 Department of Trauma and Orthopedics, Peking University People’s Hospital Qingdao Hospital, Qingdao 266111, China

**Keywords:** chronic osteomyelitis, infected bone defect, biofilm, immunomodulation, drug delivery, regenerative biomaterials, programmable therapy

## Abstract

Chronic osteomyelitis and infected bone defects are driven by recurrent infection, biofilm persistence, and dysregulated inflammation, but conventional “eradicate bacteria and fill the defect” approaches often fail to restore a regenerative microenvironment. Herein, we review biofilm-associated immune dysfunction in impaired angiogenesis/osteogenesis and summarize biomaterials that couple infection control with tissue regeneration. We integrate representative platforms into a “Time–Space–Control” framework: (i) time-programmed systems that sequence early antibiofilm/antibacterial actions with later pro-angiogenic and osteogenic cues; (ii) space-focused designs that enhance defect localization, penetration, and coverage of infected niches; and (iii) controllable strategies that enable pathology-responsive and/or externally triggered, on-demand modulation. Based on this synthesis, we propose a practical 4P principle to guide programmable therapeutic biomaterials. Overall, explicitly managing timing, localization, and controllability may improve the alignment of antimicrobial therapy, immune reprogramming, and regenerative support for chronic infected bone repair.

## 1. Introduction

**Figure 1:**
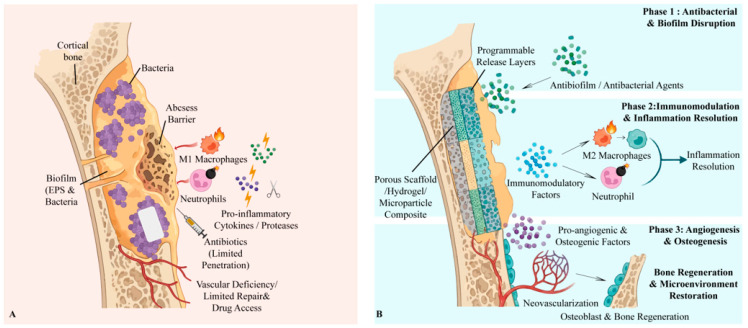
Pathology-driven spatiotemporal mismatches in chronic infected bone defects and material-enabled phase-aligned intervention. (**A**) Chronic osteomyelitis microenvironment. Biofilm-protected bacteria persist within abscess/sequestrum barriers and can colonize micro-/nano-scale bone niches, leading to poor antibiotic penetration, impaired perfusion, and sustained neutrophil/M1-macrophage-dominated inflammation that suppresses angiogenesis and osteogenesis. (**B**) Programmable biomaterial strategy. A localized scaffold/hydrogel/coating coordinates therapy across disease stages: Stage I—rapid/high-local-exposure antibiofilm/antibacterial action; Stage II—immunomodulation to resolve inflammation; Stage III—pro-angiogenic/osteogenic cues and matrix support to restore vascularization and bone formation. Created in BioRender. Jinqiu Tian. (2026) https://BioRender.com/rqeqfbw (accessed on 6 March 2026).

Infected bone defects present complex clinical failures characterized by recurrent infection, biofilm formation, chronic inflammation, and impaired healing. Even after rigorous debridement and antibiotic therapy, re-infection rates remain distressingly high in chronic osteomyelitis. Pathogenic bacteria such as *Staphylococcus aureus* persist by forming resilient biofilms and even colonizing within bone matrix channels, evading immune clearance and conventional antibiotics [1]. These biofilm sanctuaries can lead to abscess formation, wherein necrotic tissue and bacterial exopolymers create a physical barrier that prevents immune cell infiltration, making the infection self-perpetuating [2]. Moreover, persistent infection elicits prolonged inflammatory responses that disrupt normal bone healing. An unresolved influx of M1-polarized macrophages and neutrophils releases cytokines and proteases, which not only fail to eradicate the biofilm but also damage regenerative processes [3]. Compounding these issues, infected sites often suffer from poor vascularization and local immune dysfunction, further limiting tissue recovery. Current treatments are commonly focused on “kill bacteria and fill bone” and are therefore insufficient, as they do not correct the underlying microenvironmental disarray [4]. The true therapeutic goal should be to restore a healthy, well-timed healing microenvironment in regard to both space and time, rather than merely eliminating microbes and packing the defect [5,6] (Figure 1).

To tackle this challenge, an emerging shift is needed in how we design therapeutics for infected bone lesions. Instead of viewing the problem as two separate tasks, clinicians and researchers now recognize that infection control and tissue regeneration are deeply interdependent [4]. Chronic infection creates a hostile inflammatory milieu that must be modulated in tandem with antimicrobial efforts; otherwise, bone grafts or fillers will fail to integrate [7]. Traditional bone grafting or antibiotic-loaded cement spacers can fill defects and provide local antimicrobial delivery; however, they often fail to eradicate biofilm-protected bacteria and frequently exhibit non-ideal release kinetics, resulting in sub-bactericidal exposure over time. Consequently, residual infection may persist and prolong inflammation, ultimately delaying angiogenesis, osteogenesis, and defect healing [8]. Likewise, systemic antibiotics alone struggle to reach bactericidal levels inside avascular biofilm pockets and can cause systemic toxicity. Therefore, a more holistic approach is required—one that achieves spatiotemporal precision in reconditioning the defect’s microenvironment [6]. Advanced biomaterial-based delivery systems are poised to fulfill this role: by integrating antibacterial functions with osteogenic support in a single platform, together with programming their release profiles, such systems aim to transform an infected, inflammatory niche into one conducive for regeneration [7]. Overcoming infection-related bone defects demands moving beyond the simplistic “antibiotic plus bone void filler” mindset towards intelligent, environment-responsive therapeutics that can re-establish the proper spatiotemporal order of healing.

## 2. Immunopathological Mechanisms

**Figure 2:**
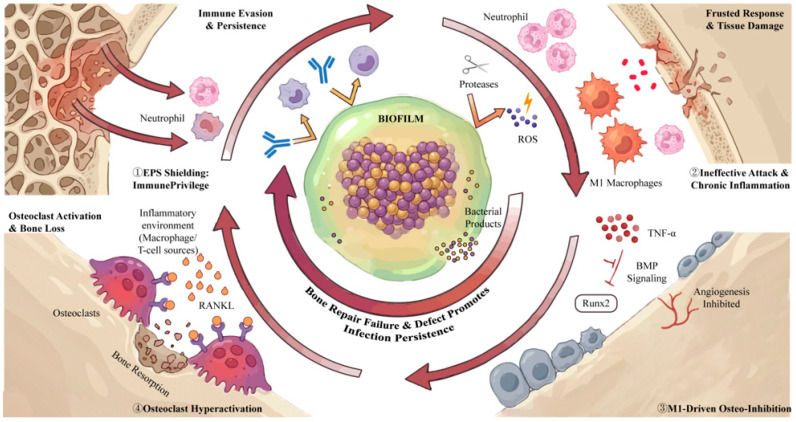
Biofilm–immunity–osteogenesis negative coupling in chronically infected bone defects. ① Immune evasion and persistence: S. aureus biofilm forms an EPS-shielded “biofilm hub,” limiting antibody/phagocyte access and enabling immune-privileged persistence. ② Aggravated immune response and tissue damage: continuous neutrophil/macrophage recruitment results in ineffective attack; ROS and proteases are released with limited biofilm clearance, aggravating local tissue injury and sustaining chronic inflammation. ③ M1-driven osteo-inhibition: persistent M1 polarization with high TNF-α disrupts angiogenesis and interferes with BMP–Runx2 signaling, suppressing osteoblast differentiation and osteogenesis. ④ Osteoclast hyperactivation and bone loss: inflammatory macrophage/T-cell–derived RANKL promotes osteoclastogenesis, increasing bone resorption and enlarging the defect, which further supports infection persistence. Created in BioRender. Jinqiu Tian. (2026) https://BioRender.com/y5njwuk (accessed on 6 March 2026).

In chronic infected bone defects, the interactions between the bacterial biofilm, the host immune system, and bone-forming cells are pathologically intertwined in a detrimental feedback loop [3]. This manifests as negative coupling among biofilm persistence, immune response, and osteogenesis. The presence of a biofilm skews and aggravates the immune reaction, and the ensuing dysregulated inflammation in turn inhibits new bone formation [2]. Bacteria within biofilms are shielded by an extracellular polymeric matrix that blocks antibodies and phagocytes, allowing pathogens to survive even amid inflammation [9,10]. Immune cells continuously recruited to the site become trapped in an ineffective battle. Neutrophils and macrophages release reactive oxygen species (ROS) and proteolytic enzymes in an attempt to penetrate the biofilm, but often to little avail. Meanwhile, the biofilm and bacterial products continuously activate immune cells, sustaining a chronic inflammatory state. This persistent M1-polarized environment is characterized by high levels of pro-inflammatory cytokines that profoundly disrupt bone regeneration. Prolonged inflammation has several deleterious effects on bone tissue. It inhibits angiogenesis, which is essential for healing; suppresses the differentiation and activity of osteoblasts; and can even stimulate osteoclastogenesis, leading to bone resorption [11]. Tumor necrosis factor-alpha (TNF-α) at chronically elevated levels can interfere with bone morphogenetic protein (BMP) signaling by promoting degradation of key transcription factors, like Runx2, thereby blocking osteoblast maturation [12]. Macrophages and T cells in the inflammatory milieu also produce excess RANKL, tipping the balance towards osteoclast activation and bone loss. As a result, instead of the normal coupled process where early inflammation transitions to healing, here inflammation and infection feed off each other and jointly impair bone repair [13]. The biofilm effectively creates an immune-privileged niche for bacteria, sometimes forming abscesses that block immune penetration, allowing the infection to persist [14]. In summary, the infectious microenvironment is locked in a vicious cycle: the biofilm drives chronic inflammation, and the inflammation prevents bone regeneration while failing to clear the biofilm. Recognizing this negative crosstalk is crucial because it means that simply killing bacteria is not enough; also modulating the immune response is necessary to break this cycle and restore conditions favorable for osteogenesis (Figure 2).

## 3. Design Principle: Time–Space–Control Axes for Programmable Therapy

**Figure 3:**
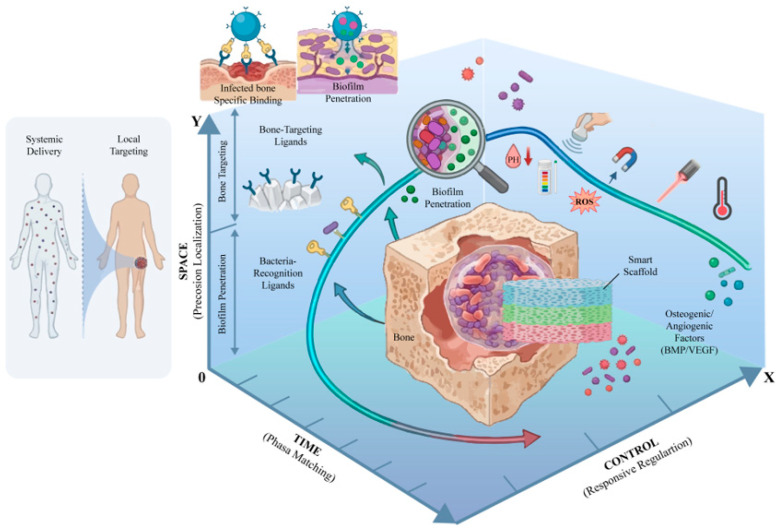
A “Time–Space–Control” coordinate system for programmable therapy in infected bone defects. Time (phase matching): a phase-aligned release profile with an early antibiofilm/antibacterial burst is followed by sustained or delayed pro-angiogenic/osteogenic delivery to avoid temporal mismatch. Space (precision localization): bone- and bacteria-targeting ligands enable defect retention, interface enrichment, and biofilm penetration, achieving high local exposure with reduced systemic spillover. Control (responsive regulation): pathology-conditional internal cues (low pH, ROS, enzymes) trigger on-site release, while clinician-applied external triggers (ultrasound, magnetic fields, light, temperature) provide on-demand, repeatable modulation for adaptable programmable release from a smart scaffold/carrier. Created in BioRender. Jinqiu Tian. (2026) https://BioRender.com/qekmwwl (accessed on 6 March 2026).

To effectively engineer a “smart” delivery system for infected bone defects, we propose a design framework defined by three key axes—Time, Space, and Control—for programmable therapy. Each axis represents a critical dimension along which the delivery strategy must be precisely tailored (Figure 3).

**Time (Temporal Axis):** Therapeutic interventions need to be phase-aligned with the progression of infection and healing. Rather than a one-shot or continuous release of all agents, the timing of drug delivery should synchronize with biological phases. For example, an early burst of antibiofilm/antibiotic action during the initial infection control phase should be followed by sustained or delayed release of osteogenic and angiogenic factors during the subsequent tissue regeneration phase. Aligning delivery with the body’s natural healing timeline ensures that each agent exerts its effect at the most opportune moment, avoiding the “time mismatch” where growth factors may be released too early into an infected, inflamed site, or antibiotics may be released too late when a robust biofilm has already formed. The temporal axis addresses when therapies are delivered, aiming for a dynamically programmed release profile that mirrors the sequential needs of the defect microenvironment [15,16].

**Space (Spatial Axis):** Therapeutic action must be place-specific, concentrated at the defect site and even within specific micro-niches of infection. This involves targeting the delivery system to bone tissue or to bacterial aggregates, thereby correcting the “space mismatch” of systemic treatments that diffuse broadly. Spatial precision can be achieved through materials and ligands that have high affinity for bone minerals or that recognize bacterial components. By localizing therapeutics to the intended site, one maximizes local efficacy (high drug concentration where needed) and minimizes off-target effects in healthy organs. The spatial axis thus covers where the therapy goes, focusing design efforts on bone-targeting, retention in the defect, and penetration into biofilms [17,18,19].

**Control (Responsive/Regulatory Axis):** This axis encompasses the mechanisms of control, that is, how the delivery system is regulated and triggered. A truly programmable system should have built-in responsiveness to relevant stimuli (internal or external) and allow on-demand modulation. Internally, this means being pathology-conditional: using infection-specific signals (pH, ROS, enzymes) as cues to automatically trigger or modulate drug release only under disease conditions. Externally, this translates to being physician-controllable: incorporating features that respond to externally applied triggers, such as temperature, light, magnetic fields, or ultrasound. Such external controls enable clinicians to actively tune the treatment post-implantation, for example, by applying a focused ultrasound pulse to trigger additional drug release during a flare-up or using a magnet to guide and concentrate nanoparticles at the defect. The control axis addresses how the release is governed, ensuring the system is not a passive dispenser but an interactive, controllable therapy that can adapt to individual patient needs or changing conditions [20,21,22].

## 4. Representative Strategies

Researchers have developed various smart delivery strategies to tackle the specific mismatches in timing, location, and patient-specific responses inherent to infected bone repair. Here, we highlight three representative categories of such strategies: segmented release systems, bone-targeting delivery, and controlled-trigger platforms (Table 1).

**Table 1:** Smart biomaterial-based therapeutic platforms mapped by a Time–Space–Control framework. The table presents studies that propose a concrete platform and therapeutic scheme (mechanistic-only, clinical cohort, and review papers are excluded). Axis (T/S/C): T, time-programmed (sequential/gradient/sustained) intervention; S, spatially targeted/localized delivery (bone/pathogen/biofilm targeting, enhanced retention/penetration); C, controlled activation/release via internal cues or external triggers (pH/ROS/enzymes, magnetic/electric field, ultrasound, NIR).

Ref#	Axis	Platform/Material	Design Logic
[15]	T	Thermosensitive CS/nano-HA hydrogel + PLGA microspheres (ball-in-ball). • CS dissolved in 0.1 M HCl; HA:CS mass ratio 1:2; β-GP for gelation. • VAN-loaded microspheres: VAN 100 mg + PLGA 250 mg. • BMP-2 microspheres: BMP-2 9 µg + PLGA 250 mg. • Reported release (30 d): VAN 57.194%; BMP-2 12.968%. • Compressive strength (CS-HA): ~1.912 kPa.	Sequential, long-horizon local delivery: early antibiotic exposure while protecting BMP-2 bioactivity for later osteogenesis; injectable in situ gel prevents systemic toxicity.
[16]	T	Thermosensitive polypeptide hydrogel (Gel) + PLGA microspheres. • Hydrogel encapsulates Gel/VA-MP or Gel/VA-MP/DGEA. • Release: VA cumulative ~91% over 48 d (reported); DGEA delayed/gradient co-release. • Inhibition zone (48 d eluate): ~20.54 mm.	Gradient/sequential co-delivery: sustained antibiotic release to sterilize + integrin-binding peptide (DGEA) to support bone repair after infection control.
[17]	S	Tetracycline-modified nanoparticles (bone-targeting) for anti-tubercular drug delivery.	Bone-homing antibiotic delivery to increase local exposure at bone lesions while reducing systemic exposure.
[18]	S + C	Gentamicin-loaded magnetic gelatin nanoparticles (GMGNPs). • Mean size: 253.7 ± 11.8 nm. • Gentamicin loading: 110.3 ± 8.2 µg/mg. • Magnetic targeting via external field.	IV magnetic drug targeting to concentrate antibiotic at infected bone; local retention + controlled release aims to improve efficacy vs. systemic dosing.
[19]	S + C	Dual-responsive pathogen-binding antibiotic-loaded nanoparticles. • Stimuli triggers: bacterial microenvironment (pH/enzymes) + binding to pathogens.	Active targeting to pathogens + on-demand antibiotic release to overcome AMR infections and reduce off-target exposure.
[20]	C	Injectable hydrogel “microsphere-bomb” (microfluidic microspheres in HAMA bulk hydrogel). • HAMA bulk: 1% HAMA + 0.5% LAP. • Microspheres: 2% EGCG + 0.2% MoS2 (EMgel). • Trigger: ultrasound (US) 1.5 W/cm^2^ for 2 min → microsphere bursting.	Externally triggered burst release + sonodynamic/catalytic synergy to eradicate MRSA in deep bone while allowing repeated, noninvasive activation.
[21]	C	Heterostructured piezocatalytic nanoparticles (Se@BTO-1). • Local injection: 300 µL at 250 µg/mL. • US activation: 1.5 W/cm^2^ for 20 min (post-op) then 0.5 W/cm^2^ for 5 min weekly.	Ultrasound-driven piezocatalysis to generate bactericidal ROS while enhancing osteogenesis; therapy is “switchable” by external US.
[22]	C	NIR-triggered implant phototherapy + immunotherapy coating on Ti (Ti-RP/PCP/RSNO). • Multihole hydrogel coating; RSNO provides NO release.	Light-activated antimicrobial phototherapy coupled with NO-mediated immunomodulation/osteogenesis to clear MRSA biofilm on implants and promote osseointegration.
[23]	T	Photo-crosslinked chitosan hydrogel (matrix) + pore-closed PLGA microparticles (fillers). • VAN in hydrogel; rhBMP-2 adsorbed into porous MPs then pore-closed by acetonitrile. • Release: VAN burst >80% in first 2 days; rhBMP-2 sustained ~12 days.	Two-compartment, time-programmed delivery: immediate antibacterial protection at early inflammation stage, then delayed growth factor to enhance osteointegration.
[24]	S	Janus-guided bone regeneration membrane (JGM): inner random gelatin fibers + nHAP; outer aligned PCL fibers + cationic copolymer P(DMC-AMA). • Electrospinning: GEL 7 wt% in TFE; nHAP 0.28% *w*/*w*.	Spatially segregated functions: bone-facing side promotes osteogenesis; soft-tissue side blocks invasion, provides contact-killing antibacterial activity and drives M2 polarization.
[25]	T	MMP-sensitive PEG hydrogel nanocomposite with covalently tethered rhBMP-2 and rhBMP-9. • Growth factors tethered (not freely diffusing);	Protease-responsive remodeling + tethered factor presentation to provide localized, long-lived osteoinductive signaling while limiting burst release.
[26]	C	Electroactive biomimetic mineralized scaffold (sp-EMS): mineralized collagen + ultrathin silver nanowires. • Ag ultrathin nanowire content: 4.81% (reported). • Porosity: 91.09%; average pore size: 117 µm. • Self-generated microcurrent: ~4 µA.	“Self-promoted” antibacterial + regenerative coupling: Ag provides rapid sterilization; electroactive microcurrent modulates immune/vascular/osteogenic responses without external power.
[27]	S + T	Bisphosphonate-conjugated sitafloxacin (BCS): bone-seeking “target & release” antibiotic prodrug. • Conjugation enables uptake/release near mineral/bone canaliculi.	Space targeting to mineralized surfaces + controlled release to reach bacteria in protected niches (e.g., osteocyte lacuna-canalicular network) where conventional antibiotics fail.
[28]	S + T	Hydroxybisphosphonate-conjugated sitafloxacin (HBCS) and comparator BCS. • Dosing: sitafloxacin 2.5 mg/kg; BCS 5 mg/kg; HBCS 3 mg/kg; vancomycin 110 mg/kg bid.	Improved bone-binding and release kinetics for eradication of established MRSA with simultaneous osseointegration after implant revision.
[29]	S	Bone-targeted delivery of cell-penetrating RUNX2 fusion protein. • Bone-targeting moiety + CPP for intracellular delivery.	Spatial targeting + intracellular delivery of transcription factor to stimulate osteogenesis (platform concept potentially portable to infection repair stages).
[30]	S + C	Aptamer-targeted liposomes for S. aureus biofilm. • S. aureus-specific aptamer (SA31) on liposome surface; MB-loaded liposomes (Apt-MBL) for photothermal adjunct. • Heat (50 °C, 10 min) enhances bacterial killing.	Biofilm/pathogen targeting via aptamer + triggered killing (heat/photothermal) to overcome biofilm tolerance.
[31]	S	Bone-targeting vancomycin-loaded liposomes. • Composition: phospholipid/cholesterol liposomes; bone-affinity ligand (alendronate) on surface.	Mineral-binding liposomes concentrate vancomycin at bone surfaces to improve local exposure and reduce systemic toxicity.
[32]	S + T	Bisphosphonate-conjugated antibiotics (carbamate-linked sitafloxacin and tedizolid). • Bone-binding bisphosphonate + cleavable linker.	Bone-targeted “prodrug” strategy to overcome pharmacodynamic limits of local therapy and improve bone penetration/retention.
[33]	C	Electroactive composite scaffold with locally expressed osteoinductive factor (electrical stimulation responsive). • Conductive composite + gene/expressed factor module.	Externally controlled electrical stimulation gates local osteoinductive signaling for synergistic repair; concept can be combined with antibacterial modules for infected defects.
[34]	T + S	ZIF-8–modified sequential-release hydrogel (SHPP-ZB). • Base hydrogel: sodium alginate + hydroxyapatite + polyvinyl alcohol (PVA). • Angiogenic cue: PDGF-BB preloaded in the hydrogel matrix. • Osteogenic cue: BMP-2@ZIF-8/PEG-NH_2_ nanoparticles embedded in the gel.	Time-programmed dual-factor delivery to accelerate vascularized bone regeneration—prioritizing angiogenic support before/alongside sustained osteogenic signaling.
[35]	T + S	Double-crosslinked hydrogel microspheres (AGMP@VEGF&BMP-2). • Fabrication: gas-assisted microfluidics to form microspheres. • Network-1: Cu^2+^ ionic crosslinking with alginate (antibacterial component). • Network-2: GelMA photo-crosslinking (structural stabilization). • Payloads: VEGF + His-tag BMP-2 co-loaded.	Integrates early infection control (antibacterial function) with subsequent pro-regenerative signaling via sequential factor presentation to support bone repair.
[36]	C + S	Photothermal–immunomodulatory nanocomposite hydrogel (PNPs@RuO_2_@HAP@Gel). • Nanophase: RuO_2_ (from Ru precursor; antioxidant/anti-inflammatory role). • Nanocarrier: polymers encapsulated with DSPE-PEG2000 → PNPs@RuO_2_ (improved dispersibility/biocompatibility). • Matrix: SBMA zwitterionic hydrogel polymerized with MBAA + APS. • Osteoconductive additive: nano-hydroxyapatite (HAP).	Employs externally triggered photothermal activation to reinforce antibacterial/biofilm clearance on demand, while concurrently modulating immune tone to support regeneration in infected defects.
[37]	T + S	Temporal immunomodulatory hydrogel (TIH). • Matrix formation: Schiff base + photo-crosslinking. • Building blocks: acrylate-modified engineered protein + oxidized sodium alginate (OSA). • Functional nanophase: zinc/calcium phosphate hybrid (ZPH) nanoparticles embedded to enable temporal ion release	Implements temporal control of immunomodulation to stage the transition from infection/inflammation control to osteogenic repair, aligning immune phase dynamics with regeneration.
[38]	S + C	Bone-targeting lipid–polymer hybrid nanoparticles (BTN). • Core: PLGA polymeric core (antibiotic encapsulation compartment). • Shell: lipid/phospholipid layer (hybrid NP architecture). • Bone-targeting ligand: alendronate (ALE; bisphosphonate) decoration for HA affinity. • Payload: antibiotic-loaded hybrid NP (MRSA bone infection application).	Bone-homing delivery to increase lesion-site accumulation and local exposure in MRSA bone infection while reducing off-target distribution, supporting less invasive, locally intensified therapy.
[39]	S + T	Vancomycin-loaded in situ gelled hydrogel (PEG/ODEX). • Matrix: polyethylene glycol (PEG) + oxidized dextran (ODEX). • Crosslinking: Schiff base reaction (aldehyde groups on ODEX reacting with amines). • Payload: vancomycin integrated into PEG/ODEX network. • Feature: injectable, in situ gelation at infection site with controlled degradability/release.	In situ gelation for defect conformability and coverage, enhancing local residence time and antibiotic exposure at infected sites to support repair of infected bone defects.
[40]	C + S + T	Photothermal-sensitive nanocomposite hydrogel with pulsed drug release (GNAG@BBR). • Matrix: GelMA/pNIPAM/pAAM composite hydrogel (thermo-responsive component included). • Photothermal unit: GO-PL (graphene oxide grafted with poly-L-lysine via EDC/NHS coupling). • Payload: berberine (BBR) incorporated for antibacterial therapy. • Feature: mild photothermal therapy + pulsed release mechanism.	Uses light-triggered photothermal control to enable reinforced/iterative antibacterial action coupled with a local matrix for defect management.
[41]	S + C	Bacteria-targeting photothermal multifunctional hydrogel (GPZC). • Matrix: GelMA + oxidized hyaluronic acid composite (GO). • Dynamic linkage: Schiff base bonds (aldehyde–amine), acid-labile in acidic environments. • Nanophase: D-cysteine-modified polydopamine nanoparticles with Zn^2+^ (PZC). • Feature: photothermal conversion (PDA) + Zn^2+^ release.	Targeting + on-demand photothermal killing with infection-microenvironment sensitivity, while Zn^2+^ supports antibacterial/osteogenic functions.
[42]	S + C	Ultrasound-triggered Mg^2+^ “blasting-release” hydrogel microspheres. • Matrix: GelMA-BP (gelatin methacryloyl-bisphosphonate) based microspheres. • Trigger module: composite nano-bubble system integrated into microspheres. • Stabilization: nano-bubble stability enhanced via metal coordination/complexation. • Trigger: ultrasonic cavitation induces nano-bubble burst → on-demand Mg^2+^ release.	Implements ultrasound-driven on-demand release to achieve controllable, potentially repeatable dosing/ion delivery for bone reconstruction; compatible with “boostable” local therapy paradigms.

### 4.1. Segmented Release Systems

Mechanism: Segmented release systems are designed to deliver therapeutic payloads in distinct phases or stages, rather than all at once, to match the evolving needs of the healing process. The core mechanism is temporal programming of drug release, which involves an initial burst release of antibiotics to quickly sterilize the area, followed by a slower or delayed release of growth factors or osteoconductive cues to support later-stage bone regeneration [23]. By partitioning the release profile, these systems ensure that each agent is active during the optimal window of healing, thereby overcoming the temporal mismatch seen in conventional one-time treatments [15].

Design Logic: The design typically involves a multi-layered or composite construct where different layers or components degrade at different rates or respond to different triggers [43]. One common approach is a bilayer or core–shell scaffold: the outer layer is loaded with antibiotic that dissolves rapidly for immediate release, while the inner core contains bone morphogenetic proteins (BMPs) or other factors in a matrix that degrades slowly [24]. Alternatively, polymers with distinct degradation kinetics can be combined [44], such as a fast-resorbing polymer carrying the antimicrobial alongside a slow-resorbing polymer carrying the osteogenic factor [25]. Some designs also incorporate a time-triggered switch, such as a hydrogel that undergoes a phase change after a certain time or enzymatic cleavage that occurs as healing progresses [45]. The idea is to program the material’s breakdown or responsiveness such that it naturally staggers the delivery of multiple agents.

Translational Perspective: Segmented release concepts have shown promising results in preclinical models of infected bone defects, demonstrating that orchestrating delivery in time indeed improves outcomes [26]. An injectable microsphere scaffold was reported to achieve >95% bacterial clearance in the first week, and then significantly enhanced bone formation by the fourth week by sequentially transitioning from an antibiotic phase to an osteogenic ion release phase [16]. Such outcomes underscore the value of phase-aligned therapy. This concept dovetails with the idea of “phase-specific” treatment: it encourages a shift in clinical protocols towards using combination products that change their activity over time, potentially reducing the need for multiple surgeries. As research continues, segmented release strategies embody the principle of phase-aligned therapy, tackling the time dimension of the problem and moving closer to controlled sequential healing.

### 4.2. Bone-Targeting Delivery

Mechanism: Bone-targeting delivery strategies are aimed at concentrating therapeutic agents specifically in bone tissue and, more precisely, at the site of the defect and infection [46]. The mechanism relies on incorporating affinity moieties or physical properties that cause the drug carrier or molecule to accumulate in bone or bind to bone mineral, thus addressing the spatial mismatch of treatments that otherwise distribute systemically. By homing in on the bone, these systems ensure higher local drug concentrations in the infected defect relative to the rest of the body [45]. This can be achieved through biochemical targeting or through exploiting physiological bone-seeking pathways [19].

Design Logic: A common design approach is to conjugate or decorate therapeutic nanoparticles/drugs with bone-affinity ligands, such as bisphosphonates (BPs). Bisphosphonates have a strong binding affinity to hydroxyapatite, the mineral in bone, and are clinically used in osteoporosis treatments [27]. By attaching a BP moiety to a drug or a nanoparticle surface, the construct will preferentially latch onto bone surfaces near the defect [28]. Another tactic is to use certain peptides or small molecules that target bone matrix proteins or collagen, guiding the payload to the fracture or defect area [29]. In addition, targeting can be directed toward bacterial markers: for instance, functionalizing a delivery system with an antibody or an aptamer that recognizes *S. aureus* components means it will bind specifically to bacteria within the bone lesion [30]. The design logic is that by endowing carriers with these targeting capabilities, one can greatly enhance drug localization: after systemic or regional administration, the carriers return to the bone defect, adhere there, and ideally even penetrate the biofilm or intracellular niches containing bacteria. This spatial targeting not only improves efficacy but also reduces systemic exposure and side effects.

Payload Combination: Bone-targeting systems can carry similar payloads as other systems, but the emphasis is on where those payloads go [46]. These strategies are used in nanoparticles or conjugates [47]. For example, a liposome or polymer nanoparticle can be loaded with antibiotic and tagged with alendronate for bone affinity [31]. They could also involve prodrugs that are activated in bone. Payloads typically include potent antibiotics, as well as bone anabolic factors if dual functionality is needed [32].

Engineering Challenges: One challenge is achieving specificity and sufficient binding. The targeting ligand should bind strongly to bone but not get “stuck” elsewhere or be cleared too rapidly. Bones in the body are widespread, so a circulating bone-targeted particle might bind to healthy bones too, not just the defect. This is beneficial for overall bone affinity, but it means one must ensure that enough of the dose reaches the target lesion. Another challenge is that the bone microenvironment can be dense. Therefore, ensuring that carriers not only stick to bone but also infiltrate the porous structure or the biofilm requires careful size and surface engineering. For bacterial targeting ligands, one must ensure they can reach the bacteria. Additionally, attaching targeting ligands can sometimes reduce a carrier’s drug loading or alter its circulation time. Manufacturing consistency is also a concern. Covalently conjugating targeting moieties in a reproducible way and at scale can be challenging. Finally, heterogeneity among patients poses a challenge: bone turnover rates and mineral content vary, and bacterial strains differ, so a targeting strategy must ideally cover broad patient variability or be customizable [48].

Translational Perspective: Bone-targeting delivery is an active area of development and holds clear translational appeal, as it aligns with the precision medicine goal of delivering drugs right to the disease site. However, since many targeting agents are themselves approved drugs, repurposing them for targeting could streamline development [28]. Bone-targeting delivery exemplifies the place-specific principle: it remedies the spatial distribution issue by ensuring that therapeutics act where the infection and bone damage reside [46].

### 4.3. Controlled-Trigger Platforms

Mechanism: Controlled-trigger delivery platforms are smart systems engineered to release therapeutics in response to specific signals or on-demand cues, thereby personalizing treatment to the patient’s condition and allowing external intervention as needed [45]. The mechanism centers on built-in sensors or triggerable components that react to either pathological stimuli or clinician-applied signals [49]. These systems remain quiescent or slow-releasing until a certain trigger is present, upon which they rapidly or additionally release their payload. This adaptability directly addresses individual heterogeneity: different patients or injury sites may have different levels of inflammation, pH, or require different timing of release [43].

Design Logic: Such platforms often incorporate stimuli-responsive materials, such as polymers or linkers that change their structure/properties when exposed to particular conditions. A hydrogel could be designed to degrade much faster in the presence of high ROS or an inflammation-associated enzyme, ensuring a surge of drug release exactly during acute flare-ups [50,51]. Alternatively, nanoparticles can be coated with pH-sensitive coatings that dissolve in acidic infection sites, or with peptides that are cleaved by bacterial enzymes, so the drug is mostly released in infected tissue and not in healthy tissue [52]. For external control, materials responsive to fields or energy are used, for example, magnetically sensitive particles that heat up or mechanically disrupt to release drugs when a magnetic field is applied [53]; gold nanorods or other photothermal agents embedded in a matrix that release drugs when exposed to near-infrared light; and acoustically responsive vesicles that open under ultrasound [33].

Payload Combination: Controlled-trigger systems can carry a variety of payloads, often similar to those in other strategies. What is distinct is how these payloads are encapsulated and linked to the responsive mechanism [54].

Engineering Challenges: Developing controlled-trigger platforms is complex. One challenge is ensuring the sensitivity and specificity of the trigger response. The system must respond robustly to pathological levels of stimulus but remain stable under normal conditions to prevent premature release [55,56].

Translational Perspective: Controlled-trigger and on-demand therapies represent a forward-looking approach aligned with personalized medicine. The benefit is clear: these platforms adapt to each patient’s dynamic condition. They embody both pathology-conditional and physician-controllable elements [56]. As such, they are perhaps the epitome of a programmable therapeutic system. When fully realized, controlled-trigger systems can significantly improve outcomes in heterogeneous patient populations by ensuring each patient effectively “gets the right dose at the right time, as determined by their own body and their physician.”

## 5. 4P Design Principle

Bringing together the insights from the above strategies, we propose a unifying 4P design principle for programmable smart delivery systems in the context of infected bone defects. The 4Ps stand for Pathology-Conditional, Phase-Aligned, Place-Specific, and Physician-Controllable. These four tenets encapsulate the essential requirements for an ideal therapeutic platform and map directly onto the challenges we have discussed.

Pathology-Conditional: The system responds to disease-specific conditions (such as infection-associated pH, ROS, enzymes), essentially releasing therapeutics conditionally upon the presence of pathology. This enables conditional payload release and minimizes off-target exposure, improving specificity for infected niches and reducing unnecessary drug burden in healthy tissue.

Phase-Aligned: The therapeutic interventions are aligned with the healing phases and timed appropriately. Rather than static or random release, systems prioritize robust early antibiofilm/antibacterial functions during infection clearance, followed by pro-angiogenic and osteogenic support during regeneration and remodeling.

Place-Specific: Therapeutics are preferentially localized to the infected defect region, achieving high on-site exposure while limiting systemic distribution. Enhanced localization and retention improve coverage of irregular defect geometries and infected micro-niches, increasing antimicrobial efficacy and supporting local tissue repair.

Physician-Controllable: Therapy remains externally adjustable, allowing clinicians to modulate dose, timing, or activation based on monitoring and patient-specific needs. On-demand control supports adaptive treatment, enables repeated activation when needed, and provides a safety lever to reduce overtreatment or manage complications. This principle is embodied in externally triggerable systems (magnetic, ultrasound, light).

These 4P elements are not isolated. They work in concert and often overlap within a single sophisticated delivery system. The ultimate vision is a therapeutic system that satisfies all 4P criteria: it releases drugs only in the diseased microenvironment (pathology-conditional), in sync with healing stages (phase-aligned), at the defect site (place-specific), and with options for doctor-controlled modulation (physician-controllable).

Importantly, the 4P principle also serves as a design language and evaluation standard. It provides researchers and clinicians with a checklist to evaluate new therapies: Does this approach activate under infection conditions? Does it deliver in a timely sequence? Does it localize to bone/injury site? Can we adjust it if needed? By formalizing these questions, the 4P framework helps ensure that future innovations do not address one aspect of the problem while neglecting others. In the context of infected bone defects, a 4P-optimized system would essentially function as a programmable therapeutic system—one that can be programmed in advance and reprogrammed in real time to orchestrate the complex healing process.

## 6. Future Perspectives

Modular and Orthogonal Design: It is possible to have separate modules for antibiofilm action, immune modulation, and osteogenesis, each triggered by different signals or on different schedules, assembled into one system. This modularity would allow personalization and flexibility. Depending on the specific clinical scenario, modules could be added or omitted. Orthogonal design means the activation of one module does not unintentionally affect the others; achieving this requires careful choice of triggers that do not cross-react.

Integrated Infection–Regeneration Metrics: As therapies shift towards the dual goals of infection eradication and tissue regeneration, there is a need for combined outcome metrics in both research and clinical evaluation. Traditional endpoints have been siloed: microbiologists focus on infection clearance (colony counts, infection recurrence), while orthopedists focus on bone healing (union rates, bone volume). In preclinical models, this may mean simultaneously measuring colony-forming units (CFU) from bone samples and bone regeneration via micro-CT or histology. Additionally, establishing standardized infection bone defect models that include both outcome measures will accelerate translational research.

Aged Model Mechanistic Assumptions: Most current research is conducted in young or healthy animal models, yet in reality, chronic bone infections often afflict older patients or those with co-morbidities. Aging and systemic health factors can significantly alter immune responses and healing capacity. It is crucial to test whether the mechanistic assumptions of smart delivery systems hold true in aged or compromised conditions. Future research should incorporate aged or disease models to validate and refine the timing and dosing logic of programmable systems.

Long-Term Safety: As we embed more “smarts” into delivery systems, including novel materials, nanoparticles, and triggers, long-term safety and biocompatibility become paramount concerns. Potential issues to monitor include chronic inflammation or foreign body response to the biomaterials, cumulative toxicity of prolonged antibiotic release, and any off-target effects of triggers. Long-term immune profiling around the implant is important, since an immune-regulating delivery might skew local immunity. Another aspect is genotoxicity or oncogenic risk. Advanced nanoparticles or persistent scaffolds should be vetted for any DNA damage potential or undue tissue hyperplasia over time. Furthermore, mechanical safety matters. Some smart scaffolds may need to degrade more slowly or maintain strength until full healing; otherwise, premature mechanical failure could re-create the defect.

## 7. Discussion

Chronic infection of bone, as seen in osteomyelitis and infected non-unions, creates a profound spatiotemporal disarray in the local tissue environment. Persistent biofilm and an enduring inflammatory state disrupt the normal timing of repair and distort the spatial distribution of pro-regenerative cues, resulting in a “loss of spatiotemporal order” that underlies recurrent infection and delayed union. Accordingly, simply combining a bactericide with a bone filler is often an oversimplification; what is needed is reprogramming of the healing process to restore coordinated transitions from infection control to vascularized osteogenesis. In this context, future delivery systems should evolve beyond static drug depots into programmable therapeutic systems capable of operating in synchrony with the patient’s healing trajectory—sensing pathological signals, delivering phase-appropriate interventions, and enabling adaptive modulation when clinical conditions change.

**Study-anchored comparison of representative biomaterial strategies:** A key insight from recent studies is that successful platforms rarely rely on a single mechanism; rather, they implement complementary functions along the Time–Space–Control axes. Representative “time-programmed” systems typically prioritize early antibiofilm/antibacterial actions and subsequently provide pro-angiogenic and osteogenic support via delayed or sustained release of growth factors, osteoinductive ions, or immunomodulators [34,35,36,37]. “Space-focused” designs improve localization and coverage of infected niches through defect-filling scaffolds, adhesive hydrogels, bone-affinitive chemistries, or ligand-mediated targeting that increases retention and local exposure while reducing systemic burden [38,39,57]. “Controllable” strategies further add pathology-responsive release and/or externally triggered actuation to enable on-demand intensification or repetition of therapy [40,41,42]. To move beyond conceptual categorization, we summarize representative platforms and their evidence levels in Table 1, mapping each system to the relevant T/S/C features. This table-based synthesis highlights practical trade-offs observed across studies (e.g., stronger antibiofilm potency sometimes coinciding with cytotoxicity risks; deeper niche penetration potentially reducing retention; externally actuated control offering flexibility but introducing workflow/equipment constraints). Collectively, the comparative view emphasizes that the field is converging on multi-functional designs that treat infection resolution and repair induction as a coordinated sequence rather than isolated tasks.

**Limitations and translational considerations:** Clinical feasibility and workflow are central: surgical debridement and dead-space management vary widely across patients, and a platform must be robust to heterogeneous defect geometry, bacterial burden, and host status while remaining simple enough for routine operative handling. Manufacturability and quality control are also major constraints, especially for multi-component systems combining antimicrobials, immunomodulators, and osteoinductive cues with stimulus-responsive elements; batch-to-batch reproducibility, sterilization compatibility, shelf-life stability, and storage conditions must be demonstrated early to support translation. Regulatory complexity increases substantially for combination products that integrate drugs, biologics, devices, and externally triggered components; clear definitions of primary mode of action, validated release/actuation specifications, and standardized performance metrics will be required. Cost and scalability must be considered in parallel with performance: platforms requiring specialized equipment or complex manufacturing pipelines may face adoption barriers unless they provide clear clinical benefit over existing standards and can be integrated into hospital workflows. Finally, safety remains a decisive gatekeeper. High local antimicrobial concentrations, reactive microenvironment-responsive chemistries, photothermal/sonodynamic effects, and persistent inorganic nanomaterials can introduce risks such as local cytotoxicity, inflammatory exacerbation, thermal injury, impaired osteointegration, or long-term residue and off-target distribution. For these reasons, translation requires rigorous biocompatibility and degradation studies, as well as careful optimization of trigger thresholds, dose ceilings, and fail-safe mechanisms.

**Gaps and future directions:** Several gaps must be addressed to strengthen the evidence base and accelerate clinical adoption. First, in vivo validation remains under-tested in clinically realistic models: many studies rely on simplified infection models or short observation windows that do not capture the recurrence-prone, biofilm-dominant nature of chronic osteomyelitis [4,5]. More standardized, reproducible models that incorporate debridement, dead-space management, and clinically relevant pathogens/biofilm formation are needed, along with endpoints that reflect both infection control and functional bone restoration. Second, there is a paucity of head-to-head comparisons against standard-of-care (SOC) regimens [57]. Without SOC benchmarking, it is difficult to quantify true incremental benefits, identify which design features matter most, and justify added complexity. Third, long-term outcomes are often missing: durable cure in COM requires demonstrating low recurrence over extended follow-up, alongside restored mechanical integrity and quality of regenerated bone. Future studies should incorporate long-term recurrence endpoints, longitudinal imaging/biomarkers, and functional/mechanical testing. Fourth, although “controllability” is frequently proposed, clinical controllability and monitorability are rarely demonstrated. Externally triggerable platforms should be evaluated under realistic constraints (penetration depth, tissue heating limits, device accessibility, repeatability) and ideally coupled with measurable feedback to enable physician-guided adjustment. Looking forward, the 4P principles proposed here can serve as both a design language and a verification standard: solutions should be pathology-conditional where possible, phase-aligned to the evolving biology, place-specific to infected niches and defects, and physician-controllable when adaptive modulation provides clear clinical value. The field has strong foundations in biomaterials, nanotechnology, and osteoimmunology, but the next step is integration—combining targeted localization, staged therapy, and controllable actuation into unified platforms—and validating these systems rigorously in clinically relevant models with long-term endpoints.

## Data Availability

No new data were created or analyzed in this study. Data sharing is not applicable to this article.

## Abbreviations

The following abbreviations are used in this manuscript:

BMP	Bone morphogenetic protein
BPs	Bisphosphonates
CFU	Colony-forming units
CT	Computed tomography
EPS	Extracellular polymeric substances
NIR	Near-infrared
RANKL	Receptor activator of nuclear factor κB ligand
ROS	Reactive oxygen species
Runx2	Runt-related transcription factor 2
TNF-α	Tumor necrosis factor-alpha
